# Prevesicular herpes zoster lumbar radiculopathy with transient motor paresis: A case report

**DOI:** 10.1097/MD.0000000000027293

**Published:** 2021-09-17

**Authors:** Kee-Won Rhyu, Jae-Hyuk Shin, Yoon-Chung Kim, Sung-Hyun Cho, Geon-Ho Kwon, Han Yong Lee

**Affiliations:** Department of Orthopedic Surgery, St. Vincent's Hospital, College of Medicine, The Catholic University of Korea.

**Keywords:** case report, dorsal root ganglion, herpes zoster, lumbar, radiculopathy, transient paresis

## Abstract

**Rationale:**

Herpes zoster frequently causes dermatomal vesicular rash accompanied by severe neuralgia, and reaching a differential diagnosis may be challenging before the appearance of the vesicular rash.

**Patient concerns:**

A 40-year-old male patient visited the emergency department with a complaint of sudden onset *motor weakness* and ipsilateral radiating neuralgia to the Lt. thigh. He had suffered from chickenpox during childhood.

**Diagnoses:**

No skin lesion was present at the initial visit. The reverse Straight Leg Raise test was negative. Magnetic resonance imaging showed asymmetrically swollen dorsal root ganglion with Gadolinium enhancement. The vesicular rash that appeared on the sixth day after the symptom onset led to the diagnosis of herpes zoster.

**Interventions:**

Antiviral agent of valacyclovir (1000 mg t.i.d.) was administered for 7 days.

**Outcomes:**

The patient recovered from *motor weaknesses* by 2 weeks from the onset of the symptom. Mild degree post-herpetic neuralgia recovered by 2 months.

**Lessons:**

A high index of suspicion is necessary to differentiate early herpes zoster radiculitis before the appearance of vesicular rash from compressive radiculopathy. In L2–3 ipsilateral radiating pain along the dermatome or myotome, the absence of reverse Straight Leg Raise sign may be a possible factor in differentiating herpes zoster radiculitis from compressive radiculopathy.

## 1 Introduction

Herpes zoster is caused by reactivation of the varicella-zoster virus (VZV), which remains latent in the sensory ganglion since its initial activation as chickenpox infection, which often occurs during childhood. Although erythematous vesicular rash and dermatomal distribution are typical symptoms during the initial radiculopathy before appearance, the vesicular rash is diagnostically challenging or confusing.^[1–4]^ Herpes zoster radiculitis may also involve motor neurons causing motor weakness along with the conventional sensory neuralgia or burning sensation.^[1]^ A few cases of herpetic lumbosacral radiculopathy before the rash eruption have been reported.^[1–4]^ Here, we present a case of a patient with an early-stage herpes zoster lumbar radiculitis accompanying motor weakness before the vesicular rash.

## 2 Case

**Figure 1:**
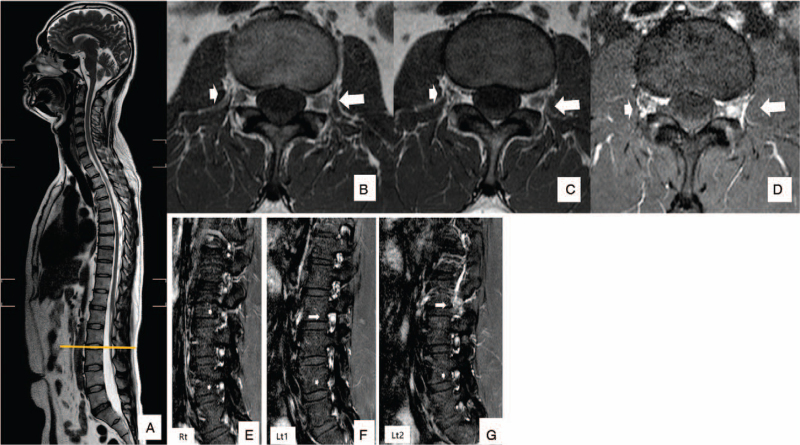
A–D, Sagittal MRI shows no definite evidence of clinical radiculopathy of the lumbar spine (A). Axial images of L2–3. Two consecutive serial axial images of L2–3 show asymmetrical thickening, or swelling of the Lt dorsal root ganglion (DRG, long arrow), compared with the contralateral DRG (short arrow) (B, C). Gadolinium enhancement revealed increased contrast enhancement of the involved (Lt) DRG (D). Sagittal image of L2–3 Lt root shows increased enhancement (long arrow, Lt1, Lt2) comparison with nerve roots of the unaffected contralateral side (E), or adjacent segments (F, G) (short arrow).

**Figure 2:**
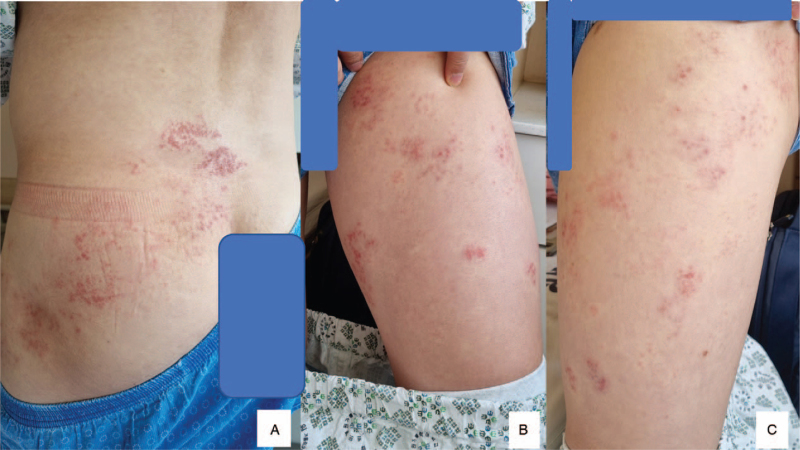
On the sixth day after the onset of radiating pain and motor weakness, the patient presented with erythematous vesicular rash, the typical symptom of Herpes Zoster (A–C).

A 40-year-old immunocompetent male patient visited the emergency department with a complaint of sudden onset severe radiculopathy to the left thigh-lower extremity for 2 days. His medical history was unremarkable for diabetes, hypertension, tuberculosis, and hepatitis. However, he had suffered from chickenpox during childhood. Radiating pain was present along the left L2–3 dermatome. Motor weakness in American Spine Injury Association Impairment Scale-Muscle Function Grading of hip flexion (V/III), knee extension (V/III), ankle dorsiflexion (V/VI), great toe dorsiflexion (V/IV), and ankle plantarflexion (V/IV) were noted. Sensory functions were intact. Babinski's sign and clonus at the ankle tests were negative. The Reverse-SLR (Straight Leg Raise) test was negative bilaterally. Lumbar spine magnetic resonance imaging (MRI) presented did not show disc herniation or compressive radiculopathy. MRI showed asymmetric swelling and Gadolinium enhancement at the dorsal root ganglion of the involved side (Fig. 1, A–G). On the sixth day of the symptom onset, the patient presented vesicular rash unilaterally on the trunk and the thigh area along the L2–3 dermatome (Fig. 2, A–C). We diagnosed the patient's condition as varicella-zoster and treated him with an antiviral agent of valacyclovir (1000 mg t.i.d.) for 7 days. The patient recovered from motor weaknesses of hip flexion, knee extension, ankle dorsiflexion, great toe dorsiflexion, and ankle plantarflexion to American Spine Injury Association Impairment Scale-Muscle Function Grading (V) by 2 weeks from the onset of the symptom. Mild degree post-herpetic neuralgia recovered by 2 months.

## 3 Discussion

VZV causes chickenpox during its initial infection, commonly during childhood. After the resolution of this primary infection, the virus remains latent in the dorsal root ganglia. Varicella-zoster is caused by the reactivation of the varicella-zoster virus after several decades because of a weakened cellular immune response. Lumbosacral radiculopathy due to herpes zoster may pose as a diagnostic challenge, especially before the rash starts to appear.^[3]^

The latent neurotropic VZV in neurons, which initially infects the cells via membrane fusion or endocytosis, is transported retrogradely inside the cell. Its reactivation involves antegrade intra-axonal transport to the synapse. Trans-synaptic transport is also reported to be a possible mechanism in brain herpes simplex virus encephalitis.^[5,6]^ Motor weakness and MRI enhancement have been shown to imply motor neuron involvement.^[7,8]^ Ante-grade intra-axonal transport from Dorsal Root Ganglion (DRG) and trans-synaptic transports through the intermediate neuron to the anterior horn cell in the spinal cord can affect the motor neuron function.^[5,9]^

The herpes virus causes acute inflammation in the sensory ganglia and the sensory gray matter of the spinal cord.^[3]^ Although it is typically diagnosed through the dermatological appearance of the vesicular rash, initial acute inflammation before the vesicular rash causes severe radiating pain, making clinicians confuse the diagnosis with compressive radiculopathy. Motor nerve root involvement has been suggested to cause motor weakness.^[1,10]^ An increasing number of herpes zoster radiculopathy cases have been reported in the past.^[2]^ A selective nerve root block injection may help relieve radiculopathy symptoms. However, beyond clinical symptom relief, scientific evidence elucidating the mode of action of this treatment method remains to be investigated.^[3]^

Teo et al^[8]^ reported a case of a 74-year-old patient with comorbidities of diabetes mellitus, hypertension, and ischemic heart disease. He presented with motor weakness of unilateral hip flexor weakness (Gr III) with L2–3 hyperaesthesia on the 5 to 6th day of the symptom onset, simultaneously showing motor weakness and the vesicular rash, which helped bypass clinical observation for the diagnostic ambiguity. His MRI findings were unremarkable at presentation. Bhushan et al^[1]^ reported acute lumbar herpes zoster radiculopathy with MRI enhancement to the L5-DRG and the nerve root; however, their patient did not present corresponding motor weakness on L5/S1 myotome. Changa and Jain^[7]^ reported a case of a patient with posteruption herpes zoster radiculitis accompanied by motor weakness and MRI enhancement along the extraforaminal nerve root. Some patients may also present with lumbar-postsurgical herpes zoster radiculitis, alerting differentiation from a postsurgical radiating pain.^[4]^ These similarities in symptoms present several challenges in reaching a differential diagnosis between neuropathic vs compressive radiculopathies before the presentation of the vesicular rash in zoster.

Sequelae from persistent herpes zoster virus reactivation may lead to demyelination, blindness, facial paralysis, or motor sequelae of foot drop.^[11–15]^ Postmortem brain study of 37 patients diagnosed with multiple sclerosis using polymerase chain reaction and Southern blot hybridization showed a higher prevalence of herpes simplex virus-1, and -2 infection compared with controls.^[16]^ Based on these reports, it can be hypothesized that persistent herpes virus reactivation causes neural demyelination, directly or by immune-mediated scar formation, eventually causing functional neural loss, markedly in the narrow nerve endings such as the ophthalmic nerve or the facial nerve. Early antiviral treatment with proper management may help reduce the risk of motor paralytic sequelae. Clinical relevance regarding herpes virus reactivation in the DRG and the spinal cord remains to be further investigated. Our patient presented Hip flexion (Grade III) motor weakness, knee extension (Grade III), and sensory neuralgia to the left thigh at the initial onset of the symptom. Initial physical examination of reverse SLR test after suspecting upper lumbar herniation of nucleus pulposus was negative. Lumbar spine MRI evaluation showed no definite evidence of compressive neuropathy corresponding to the symptom. However, asymmetric swelling of the DRG at the L2–3 intervertebral segment was noted with asymmetric Gadolinium contrast enhancement (Fig. 1, A–G).

Interestingly, while the patient was initially suspected of upper lumbar disc herniation (eg, L2–3), the patient showed no sign of reverse SLR symptom, as indicated by the absence of compressive radiculopathy on MRI. Our patient with early herpes zoster radiculopathy exhibited a combined presentation of radiculopathy before the rash, presence of motor weakness, asymmetric DRG swelling and enhancement, and motor recovery through antiviral medication. The long-term consequences of the repetitive latent herpes neuritis on the neural tissue or function are not fully understood. The preventive effect through medication needs to be further investigated.

In conclusion, we recommend that clinicians should maintain a high index of suspicion to differentiate early herpes zoster radiculitis from compressive radiculopathy before the vesicular rash appearance. In L2–3 ipsilateral radiating pain along the dermatome or myotome, the absence of reverse SLR sign may possibly help differentiate herpes zoster radiculitis from compressive radiculopathy.

## Acknowledgments

The authors thank Editage (www.editage.com) for English language editing.

## Author contributions

**Conceptualization:** Kee-Won Rhyu, Jae-Hyuk Shin.

**Data curation:** Jae-Hyuk Shin, Sung-Hyun Cho, Geon-Ho Kwon.

**Investigation:** Kee-Won Rhyu, Jae-Hyuk Shin, Yoon-Chung Kim, Sung-Hyun Cho, Geon-Ho Kwon, Han Yong Lee.

**Supervision:** Kee-Won Rhyu, Jae-Hyuk Shin, Han Yong Lee.

**Validation:** Yoon-Chung Kim, Sung-Hyun Cho, Han Yong Lee.

**Writing – original draft:** Jae-Hyuk Shin.

**Writing – review & editing:** Jae-Hyuk Shin, Yoon-Chung Kim, Sung-Hyun Cho.
